# Early storybook reading and childhood development: A cross-sectional study in Iran

**DOI:** 10.12688/f1000research.14078.1

**Published:** 2018-03-29

**Authors:** Firoozeh Sajedi, Elham Habibi, Nikta Hatamizadeh, Soheila Shahshahanipour, Hosein Malek Afzali

**Affiliations:** 1 Pediatric Neurorehabilitation Research Center, University of Social Welfare and Rehabilitation Sciences, Tehran, Iran; 2 International Child Neurology Association, Cape Town, South Africa; 3 Department of Epidemiology and Biostatistics, School of Public Health, Tehran University of Medical Sciences, Tehran, Iran

**Keywords:** Book Reading, Child Development, Early Interventions

## Abstract

**Background** **:** Development is a process that continues from childhood to death, and most developmental changes occur during childhood. UNICEF introduced early storybook-reading (ESR) and storytelling as part of child care indicators. The aim of this study was to investigate the status of book-reading to children and its relationship with early childhood development in Iran.

**Methods:** This is a descriptive-analytic study conducted in Tehran April-May 2017. In total, 272 mothers of children aged 3-30 months, who were referred to health centers, were selected using a convenience sampling method. Exclusion criteria was scoring below the cutoff point of any developmental domains of the Ages and Stages Questionnaire (ASQ). ESR was assessed by checklist and child development was assessed by the ASQ. Data were analyzed using SPSS.

**Results:** The mean number of children’s books owned was 10.23±8.642, and 84.75% had at least 3 books. The average book reading, storytelling and singing duration for children was 10±9.65, 11.48±11.756, and 23.88 ±17.880 min per day, respectively. Average book reading, storytelling, and singing duration was significantly greater in children 18-30 months than <17 months. There was a significant relationship between the number of books and a child's age, mother's age, family income, income satisfaction, father's employment, and parents’ education. The score of communication domain in the ASQ questionnaire was significantly related to the number of books, duration of reading and storytelling, while problem-solving had a significant relationship only with the number of books (p˂0.05). Based on linear regression, child's age, income, and mother's and father's educational level were models for predicting the number of children's books (p=0.0001 for all).

**Conclusions:** ESR was associated with some developmental domains of communication and problem-solving in the present study. Therefore, creation of ESR culture in Iranian families as an integral part of the life of children is necessary from birth.

## Introduction

Development is a complex process through which an individual acquires various capabilities to improve performance and have better adaptation to the environment. Although, this process continues from childhood to death, most developmental process occurs during the first few years of life ^1^, during which the neural structure evolves ^2^. Although the opportunity for early childhood interventions is short, it has significant results ^3^, and it is believed that a better beginning of life will lead to a better future ^4^. Therefore, providing the best beginning for children can lead to success in life, employment, higher earnings, active participation in society, increased responsibility, and reduction of crime and some chronic diseases ^5, 6^. The prevalence of developmental disorders in Iranian children is between 7 and 22.4% ^7^ and is reported to be 14–20% in other countries ^8– 11^. However, by encouraging mothers and community participation, and timely and appropriate intervention in controlling the risk factors, the ability to promote development, health, well-being and suitability of the child can also be provided ^12– 14^.

Many social factors and environmental stimuli are effective in health and early childhood development (ECD) ^12^, which include useful books and toys that provide a healthier life in the long run for the individual. Book reading is regarded as one of the most powerful protections for mental growth and development, and psychological and social stimulation of a child at home ^15, 16^. UNICEF introduced early storybook reading (ESR) and storytelling as child care indicators ^16^. In addition, the parent-child's common interest and joint attention in reading picture books provides the basis for social and emotional communication during childhood. Reading books to children promotes their development of speech and language, and cognitive and emotional social behavior ^17– 19^, and provides them with joy ^20^. In addition, the infant learns how to take the books in their hands and try to turn its pages. Hence, it can promote gross and fine motor coordination and also a love for reading ^21^. When book reading is started in early months of life, a longer and more stable effect on child development will be observed ^22^, as reported by Senechal and LeFevre ^23^ who found that parents should begin book reading to infants at the age of 4 months, since these children had better literacy skills at school age. It is believed that ESR promotes the development of children; Murray and Egan ^24^ reported a positive relationship between reading books and the score of cognitive development in 9-month-old infants. Cline and Edwards stated that ESR in addition to enhancing child's development and learning skills, also creates an interest, motivation and habit of regular book reading in the individual in the future ^22^. Therefore, it is good for all family members to participate in book reading to children, and children’s books should be made available for them at home ^22^. Additionally, during reading, a warm and intimate interaction between adults, especially parents and the children is created, and along with playing, can create love and reading infrastructure for motivation in later years ^22, 25^. In addition to book reading, storytelling and singing also have similar benefits in children and can be done anywhere or any time ^22^. It is believed that ESR, storytelling and singing to children are a series of activities that help in the development of speech and language, literacy and children's brain development ^26^. The most important ESR barriers are inadequate access to children's book ^27^, and parents' lack of awareness on the positive effects of ESR and inappropriate strategies for promoting children development ^27– 29^.

In general, few studies have been conducted on the role of children's books in the lives of children under the age of three ^30^. Reading books to children is considered as an important activity in Western culture ^31^, including the BookStart program in the United Kingdom, which over two decades implemented reading books in the early years of life has as an Early Interventions (EI) ^32^. Although there have been numerous studies on ECD in Iran, reading to young children is not considered as one of the environmental stimuli affecting ECD. Accordingly, this study aimed to examine the status of book reading on Iranian children, including children's books, parents' participation in reading, storytelling and singing to children based on ECD in infants and toddlers without any developmental delay during early childhood in Tehran.

## Methods

### Study design and setting

This is a descriptive-analytic cross-sectional study that aimed to investigate the status of book reading on young children and its relationship with ECD in Tehran from April to May, 2017.

The research population consisted of all mothers with children from 3 to 30 months, who were referred to one of the public health centers in Tehran for various health-related services. For sampling, Tehran was divided into two regions, East and West, and one health center was selected from each of the two regions randomly Ershad and Shahre Ziba public health centers were selected in the East and West of Tehran, respectively. Sampling of mothers was conducted using convenience sampling method. Mothers that were referred to the health center for a routine checkup for her child, child vaccination and/or receiving other health services, were entered in the study.

### Participants

Using a sample size formula ^1^ and a 95% confidence level, and also considering the prevalence of developmental disorders, which was considered to be about 22% in Iran in accordance with previous studies ^7^, with an increase of 10% to take account of potential attrition, 290 people were enrolled in the study, from which 272 consented to participate.

The criteria for entry in this study were parents with a child aged 3 to 30 months who, while referred to the health centers, who were willing to cooperate. At first, the aims of the study were explained to the parents once they were referred to the health center. They also provided written informed consent and completed self-report questionnaires in about 10–15 minutes. The mothers of children with developmental delay (based on ASQ) were excluded. 

### Assessment tools

The assessment tools of this study were the ASQ, demographic questionnaire and a checklist for assessing the ESR status of children ( Supplementary File 1). The demographic questionnaire and checklist were prepared by the research team and validated by 7 pediatricians and specialists in child development who worked in University of Social Welfare and Rehabilitation Sciences of Iran. These tools were piloted by 8 mothers that were referred to the Saadat Abad Health Center. This checklist included the number of children's books, duration of reading, storytelling and singing for children as variables.

The ASQ is used to screen infants and young children for developmental delays during the crucial first 5 years of life. This tool assess the different developmental domains (gross motor, fine motor, communication, problem-solving, and social-personal) ^33^. For each age group, a total of 30 questions (six questions for each developmental domain) were designed and the highest score available for each question is 10; therefore for each developmental domain, the score is 60. The answer to each question included yes, sometimes and never with 10, 5 and 0 points considered for each question, respectively. This questionnaire is a standard tool that has been translated into various countries in Asia, Europe, Africa and the United States ^34, 35^, and the sensitivity and specificity of this test are 88% and 82.5%, respectively ^35, 36^. The screening of developmental delay was done by comparing the score of each area with a cut-off point ^37^ based on the age of Iranian children. According to a study by Sajedi *et al.* ^38^ the Cronbach's alpha obtained was 0.79 and, the validity, reliability, and ability of the test for determination of evolutionary deficits were 0.84, 0.94 and 96%, respectively ^37^. As it is possible that parents may rate their children more favorably, and the ASQ is a self-report questionnaire, the mothers were given adequate explanations of each question and answer before completing the questionnaires in order to reduce reporting bias.

### Data analysis

Due to the nature of the present study, demographic data, ESR status for children, and ASQ questionnaire scores were standardized in terms of mean and standard deviation. Categorical data were summarised using absolute values (percentage). After determining the data normality of each variable, Chi-square, independent t-test, and correlation coefficients Spearman’s test were used. Considering the fact that the data gathering tools (i.e. the questionnaire) was a self-reported one, despite of all counter measures, having missing data was inevitable. Assessment of the impacts of such missing data, was made by Univariate T-Test. The results revealed that the missing data were completely at random ^39^. In order to predict the situation of ECD, independent variables were included in the linear regression model. Data were analyzed by SPSS software version 22 at a significant level of p<0.05.

### Ethical statement

The present research was approved by the University of Social Welfare and Rehabilitation Sciences (ethics code IR.USWR.REC.1395.77). This study observed ethical standards, and after sampling, in the case of results from the ASQ showing probability of developmental delay for certain children, this was discussed with pediatricians who were in contact with parents.

## Results

The number of mothers included in the study was 272. Most of the children (86.86%) were born by cesarean section method, 50.9% were girls, and 21.5% were also cared for by grandparents in addition to their parents. As shown in Table 1, the mean age of mothers and fathers was 31.72 ± 4.450 and 35.5 ± 5.149 years, respectively. 68.4% of mothers were housewives and 5.5% of fathers were unemployed. The average income of families was 31,150,000 Rials, 54.8% of whom were satisfied with their income. 73% of children did not have any siblings.

**Table 1.** Demographic characteristics of the family according to age group of the infants and toddlers.

	Age group (months)		
Variables, mean±SD (range)	3 to 17 (N=165)	18 to 30 (N=107)	Total (N=272)
Infants age (months)	9.81±4.022 (3-17)	23.83±3.886 (18-30)	15.33±70924
Birth weight infant (g)	3161±383 (2200-4300)	3063±423 (1950-4100)	3.122±0.401
Gestational age (weeks)	37.31±1.987 (29-41)	36.72±2.462 (28-41)	37.04±2.168
Mothers age (years)	31.55±4.443 (23-42)	31.97±4.469 (21-43)	31.72±4.450
Fathers age (years)	35.09±5.060 (25-49)	36.32±5.218 (25-50)	35.58±5.149
Mother’s education (N, %) *Diploma* *Academic*	28 (17.0) 137 (83.0)	24 (22.5) 83 (77.5)	52 (19.1) 220 (80.9)
Father’s education (N, %) *Diploma* *Academic*	44 (26.6) 121 (73.4)	34 (31.7) 73 (68.3)	78 (28.7) 194 (71.3)
Mother’s job (N, %) *Employed* *Housewife*	59 (35.8) 106 (64.2)	27 (25.9) 80 (74.1)	86 (31.6) 186 (68.4)
Father’s job (N, %) *Employed* *Unemployed*	153 (92.7) 12 (7.3)	104 (97.2) 3 (2.8)	257 (94.5) 15 (5.5)
Income (Rial, monthly)	30,380,00±1,483,000 (3,000,000-80,000,000)	32,280,000±2,252,800 (4,000,000-180,000,000)	31,150,000±1,830,000
Satisfaction of income (N, %) *Satisfied* *Unsatisfied*	84 (50.9) 81 (49.1)	65 (60.7) 42 (39.3)	149 (54.8) 123 (45.2)

On average, every child had 10.2±8.642 books. Among the children, 30 (11.20%) did not have any books and 228 (84.75%) had at least 3 books. Only five children (1.83%) had more than 37 books each. The average daily reading time for children was 9.65 ± 10, while for half of them, nobody read 5–15 min to them a day, and 27.6% of them were never read to. The average storytelling time for children was 11.48 ± 11.756 min per day, while 25.7% of parents had never told the children a story. The average duration of singing for children was 23.88 ± 17.880 min per day, but for 11.4% of them, nobody has ever sung.

According to the results in Table 2, the number of children's books owned, duration of ESR, storytelling and singing in children was significantly different between children aged 3 to 17 months and those aged 18–30 months (p=0.0001 for all). There was a significant relationship between the number of children’s book owned with duration of ESR, storytelling and singing (p<0.0001). In addition, there was a significant association between the number of children's book owned and children's age (p<0.0001), mother's age (p=0.021), family income (p=0.009), income satisfaction (p=0.026), father's occupation (p=0.04), and mother's and father's educational level (p<0.0001). ESR duration for children was significantly correlated with the age of the child, mother and father (p<0.0001). Singing duration was significantly correlated with only the mother's educational level (p=0.011).

**Table 2.** Early Storybook Reading situation of infants and toddlers.

	Age group (months)		
Variables, mean±SD (range)	3 to 17 (N=165)	18 to 30 (N=107)	Total (N=272)
Amount of children's books owned *No books owned (number of children, %)*	8.02±7.713 (0-40) *25 (15.2)*	13.57±8.929 (0-45) *5 (4.7)*	10.23±8.642 (0-45) *30 (11)*
Duration of book reading (min per day) *No book reading (number of children, %)*	7.42±8.571 (0-30) *61 (37)*	13.04±11.058 (0-60) *14 (13.5)*	9.65±10.001 (0-60) *75 (27.6)*
Duration of storytelling (min per day) *No storytelling (number of children, %)*	9.93±12.395 (0-60) *60 (36.4)*	13.88±10.592 (0-60) *10 (9.3)*	11.48±11.856 (0-60) *70 (25.7)*
Duration of singing (min per day) *No singing (number of children, %)*	22.49±18.4996 (0-90) *25 (15.2)*	24.76±16.872 (0-60) *6 (5.6)*	23.38±17.880 (0-90) *31 (11.4)*

The correlation between ESR variables is shown in Table 3. From the viewpoint of development, the scores of communication and problem-solving from the ASQ questionnaire showed a significant relationship with the number of children's books owned (p<0.0001 and p=0.016). Also, there was a significant correlation between the score of communication and duration of ESR and storytelling (p=0.002 and p=0.028). There was no significant relationship between variables and duration of singing (P>0.05 for all variables). Based on linear regression, children's age, income, and mother's and father's educational level can be models for predicting the number of children's books owned (p<0.0001 for all).

**Table 3.** Correlation between Early Storybook Reading (ESD) and Early Childhood Development (ECD).

ESD Variables Domains of ECD, mean±SD N=272)		Number of children’s books owned	Duration of book reading (min per day)	Duration of storytelling (min per day)	Duration of singing (min per day)
Communication (48.70±9.742)	Spearman Correlation Sig(2-Tailed)	0.279 ^*^ 0.000	0.136 ^*^ 0.028	0.191 ^*^ 0.002	0.169 0.066
Gross Motor skills (51.76±8.315)	Spearman Correlation Sig(2-Tailed)	0.113 0.065	0.094 0.126	0.094 0.126	0.096 0.117
Fine Motor skills (50.02±9.161)	Spearman Correlation Sig(2-Tailed)	0.096 0.115	0.059 0.345	0.014 0.825	0.119 0.053
Problem-Solving (52.34±7.010)	Spearman Correlation Sig(2-Tailed)	0.147 ^*^ 0.016	0.099 0.110	0.039 0.532	0.089 0.149
Personal-Social (48.68±9.457)	Spearman Correlation Sig(2-Tailed)	0.066 0.279	0.040 0.514	0.074 0.231	0.097 0.115

*Correlation is significant at the level 0.05 (2-Tailed).

ESR and ECD data from the East and West of Tehran Click here for additional data file. Copyright: © 2018 Sajedi F et al. 2018 Data associated with the article are available under the terms of the Creative Commons Zero "No rights reserved" data waiver (CC0 1.0 Public domain dedication).

## Discussion

In the present study, the average number of children’s books owned was 10.23±8.642, which increased significantly in infants aged 18 to 30 months as compared to infants less than 17 months old (p=0.0001). In other studies, this number was reported to be 2.6± 3.600 to 7.9± 10.9 for children aged 6 to 18 months, indicating an increase in the number of children's books owned based on the age of the children, and it is believed that during early infancy, playing is considered more important than book reading ^30^. A total of 84.75% of the children had at least 3 children’s books; this was reported to be 97% in Ukraine and 3% in Laos ^16^. According to the results of the current study, more than 68% of the parents had a university education, although in Shahshahani *et al.’s* ^40^ only 34% of parents had a university education. In the present study, there was a significant relationship between the number of books and the duration of ESR with parental education, while in this case Boyle and colleagues believed that educated mothers used better medical education recommendations ^41^, while fathers with university education can provide a more sustainable financial and social environment for their children ^21^. In addition, mother's level of education plays a facilitating role in the book reading of children and ECD ^21^. According to some similar studies, educated parents also read more books to their children ^42^. However, in the study by Tomopoulos *et al.* ^30^, maternal education was not associated with ESR. The mean ESR time in the current study for children aged less than 17 months and from 18 to 36 months was 7.42±8.571 and 13.04±11.058 minutes per day, while in the study of 30, the mean for the ages of 6 and 18 months was 2.1±2.300 and 3.5±2.800 days per week, respectively. Although in the present study, father's employment was significantly correlated with the number of children's books owned; there was no significant relationship between father's occupation and ESR, which is similar to result presented in 30.

Given the average number of books in this study, for 61.4% of children aged 0–17 months, book reading was begun at the time of sampling; this statistic increased to 86.5% in children aged 18 to 30, while based on the results of Duursma ^21^, book reading was done daily or weekly for 77% of the children who were 14 and 24-months-old, 52% of children aged 4 to 35 months ^43^ and 50% of children aged 0 to 36 months ^27^. According to Senechal and LeFevre ^23^, the number of picture books in a home has a strong relationship with the children's receptive and expressive language. Therefore, parents should be advised to read books to their children at the earliest opportunity, from birth, because it can be effective in their development ^5^. Meanwhile, in the current study, 28.6% of the children had never been read to, while in other studies, this statistic was 4% in the UK ^27^ and 6–23% in the USA ^21, 43^.

According to the present findings, family income showed a significant relationship with the number of books owned and duration of ESR. In this regard, Karrass and his colleagues ^44^ also found in 2013 that mothers of higher income families had a greater tendency for ESR for their 8-month-old infants. Additionally, according to a UNICEF report ^16^ in 2012, children from poor families had fewer children’s books at home.

In the present study, 62.5% and 90.4% of children aged 0–17 and 18 to 30 months were told stories. Also, 84.6% and 94.24% of children aged 0–17 months and 18 to 30 months were sung to, indicating an increase in these stimuli with the age of the child. In confirmation, other researchers also believe that storytelling and singing to children helps in speech evolution, language and literacy, as well as the evolution of the baby's brain ^45^.

Having a book and reading it at home will provide more opportunities for improving skills ^46^. According to the results of the current study, there was a positive correlation between the number of children's books owned and problem-solving scores. In confirmation of this result, by using ASQ, Murray and Egan’s research also found that reading books to infants had a positive impact on problem-solving and communication issues ^11^. In this regard, Duursma ^21^ also proposed the ability to predict 24-month cognitive skills score by Bayley MDI based on the time spent in reading to a child. Also, loud book reading had a significant effect on the cognitive development of premature infants after two years ^47^. In the present study, there was a positive correlation between the number of children's books owned, the duration of ESR and storytelling for children and the communication score. In relation to the impact of ESR on speech-language development, results of research indicates that children use a rich vocabulary during book reading ^18, 31^ and interventions for the development of the children's language, focusing on reading books whenever possible in children's lives are required ^48^. However, in the current research, there was no relationship between ESR and other developmental domain scores, but in another study it was reported that ESR may promote language and social communication skills ^49^.

### Limitations

In this study, the important environmental stimuli (having a book at home and its number, and duration of book-reading, storytelling and singing) in children without any developmental delay in Tehran, was described, thus there was no information about ESR and its effect on children with developmental delay. Therefore, it is suggested that, in addition to conducting more extensive studies in the country, the quality and style of book reading for children should be addressed in the future in Iran.

## Conclusion

According to the results of this study, ESR is considered as an early environmental stimulus and interventions in domains of communication and problem-solving in children. Therefore, the establishment of ESR culture among families and individuals in the Iranian community as a recreational and inseparable part of children's life from birth is recommended. As a result, due to the differences between culture and traditions among Iranian people, it is suggested more extensive studies be conducted on the quality, style of reading, and the views of parents and other caregivers on EI when dealing with reading books to children and its effects on ECD in Iran. The results of this study are only generalizable to Iranian children of Tehran.

## Data availability

The data referenced by this article are under copyright with the following copyright statement: Copyright: © 2018 Sajedi F et al.

Data associated with the article are available under the terms of the Creative Commons Zero "No rights reserved" data waiver (CC0 1.0 Public domain dedication). 

Dataset 1: ESR and ECD data from the East and West of Tehran. Variables are coded as follows: Birth rank: 1=The first child, 2=The second child and more; Caregivers: 1=mother, 2=father, 3=grandparents, 4=others; Age group: 0=3 to 17, 1=18 to 30; New Gestational age: 1<37 Week, 2≥37 Week; New mother educate: 1=diploma and below, 2=academic; New father educate: 1=diploma and below, 2=academic; New mother job: 1=Employed, 2=Housewife; New father job: 1=Employed, 2=Unemployed. DOI, 10.5256/f1000research.14078.d198657 ^50^

## Formula

^1^ $n=\;\frac{\left(Z_{1–\frac{\alpha }{2}})2\times P(1–P\right)}{d^{2}}$

## Supplementary material

Supplementary File 1: Survey of demographic and ESR in Persian with an English translation.

## References

[1] Bhattacharya T Ray S Das DK: Developmental delay among children below two years of age: cross-sectional study in a community development block of Burdwan district, West Bengal. *Int J Community Med Public Health.* 2017;4(5):1762–7. 10.18203/2394-6040.ijcmph20171798

[2] O'Mahony SM Clarke G Dinan TG: Early-life adversity and brain development: Is the microbiome a missing piece of the puzzle? *Neuroscience.* 2017;342:37–54. 10.1016/j.neuroscience.2015.09.068 26432952

[3] Schady N Behrman J Araujo MC: Wealth gradients in early childhood cognitive development in five Latin American countries. *J Hum Resour.* 2015;50(2):446–63. 10.3368/jhr.50.2.446 25983344 PMC4431591

[4] Phajane MH: The Quality Care and Education of the Child: The Unspoken Realities. *International Journal of Educational Sciences.* 2016;12(2):106–12. 10.1080/09751122.2016.11890417

[5] Hasford J Loomis C Nelson G: Youth narratives on community experiences and sense of community and their relation to participation in an early childhood development program. *Youth Soc.* 2016;48(4):577–96. 10.1177/0044118X13506447

[6] Woodhead M Ames P Vennam U: Equity and quality? Challenges for early childhood and primary education in Ethiopia, India and Peru.Working Paper.2009 Reference Source

[7] Sajedi F Doulabi MA Vameghi R: Development of Children in Iran: A Systematic Review and Meta-Analysis. *Glob J Health Sci.* 2016;8(8):51251. 10.5539/gjhs.v8n8p145 27045395 PMC5016360

[8] Bhasin TK Brocksen S Avchen RN: Prevalence of four developmental disabilities among children aged 8 years--Metropolitan Atlanta Developmental Disabilities Surveillance Program, 1996 and 2000. *MMWR Surveill Summ.* 2006;55(1):1–9. 16437058

[9] Boyle CA Boulet S Schieve LA: Trends in the prevalence of developmental disabilities in US children, 1997–2008. *Pediatrics.* 2011;127(6):1034–42. 10.1542/peds.2010-2989 21606152

[10] Silove N Collins F Ellaway C: Update on the investigation of children with delayed development. *J Paediatr Child Health.* 2013;49(7):519–25. 10.1111/jpc.12176 23600797

[11] Sutter-Dallay AL Murray L Dequae-Merchadou L: A prospective longitudinal study of the impact of early postnatal vs. chronic maternal depressive symptoms on child development. *Eur Psychiatry.* 2011;26(8):484–9. 10.1016/j.eurpsy.2010.05.004 20621453

[12] Gulland A: Health inequalities are worsening across Europe, says WHO. *BMJ.* 2013;347:f6594. 10.1136/bmj.f6594 24179086

[13] Roshanfekr P Gharibzadeh S Mohammadinia L: Involving mothers in child development assessment in a community-based participatory study using ages and stages questionnaires. *Int J Prev Med.* 2017;8(1):102. 10.4103/ijpvm.IJPVM_268_17 29291044 PMC5738788

[14] Vameghi R Sajedi F Bandpei Mohseni MA: Motor developmental delay in 7500 Iranian infants: Prevalence and risk factors. *Iran J Child Neurol.* 2009;3(3):43–50. 10.22037/ijcn.v3i3.1468

[15] Aboud FE Yousafzai AK: Global health and development in early childhood. *Annu Rev Psychol.* 2015;66:433–57. 10.1146/annurev-psych-010814-015128 25196276

[16] Unicef: Inequities in Early Childhood Development: What the data say: Evidence from the Multiple Indicator Cluster Surveys.New York: UNICEF.2012 Reference Source

[17] Mol SE Bus AG: To read or not to read: a meta-analysis of print exposure from infancy to early adulthood. *Psychol Bull.* 2011;137(2):267–96. 10.1037/a0021890 21219054

[18] Hoff E: Context effects on young children’s language use: The influence of conversational setting and partner. *First Lang.* 2010;30(3–4):461–72. 10.1177/0142723710370525

[19] Council NR: Preventing reading difficulties in young children.National Academies Press; Washington, DC.1998 Reference Source

[20] Sénéchal M LeFevre JA Hudson E: Knowledge of storybooks as a predictor of young children's vocabulary. *J Educ Psychol.* 1996;88(3):520–536. 10.1037/0022-0663.88.3.520

[21] Duursma E: The effects of fathers' and mothers' reading to their children on language outcomes of children participating in early head start in the United States. *Fathering.* 2014;12(3):283 Reference Source

[22] Cline KD Edwards CP: Parent–child book-reading styles, emotional quality, and changes in early head start children’s cognitive scores. *Early Educ Dev.* 2017;28(1):41–58. 10.1080/10409289.2016.1177392

[23] Sénéchal M LeFevre JA: Parental involvement in the development of children's reading skill: a five-year longitudinal study. *Child Dev.* 2002;73(2):445–60. 10.1111/1467-8624.00417 11949902

[24] Murray A Egan SM: Does reading to infants benefit their cognitive development at 9-months-old? An investigation using a large birth cohort survey. *Child Lang Teach Ther.* 2014;30(3):303–15. 10.1177/0265659013513813

[25] Sosa AV: Association of the Type of Toy Used During Play With the Quantity and Quality of Parent-Infant Communication. *JAMA Pediatr.* 2016;170(2):132–7. 10.1001/jamapediatrics.2015.3753 26720437

[26] Council on Early Childhood, High PC Klass P: Literacy promotion: an essential component of primary care pediatric practice. *Pediatrics.* 2014;134(2):404–9. 10.1542/peds.2014-1384 24962987

[27] Fletcher KL Reese E: Picture book reading with young children: A conceptual framework. *Dev Rev.* 2005;25(1):64–103. 10.1016/j.dr.2004.08.009

[28] Habibi E Sajedi F Afzali HM: Early Childhood Development and Iranian Parents' Knowledge: A Qualitative Study. *Int J Prev Med.* 2018;8:84. 10.4103/ijpvm.IJPVM_159_17 29142650 PMC5672654

[29] Sajedi F Habibi E Malek Afzali H: An approach towards promoting Iranian caregivers’ knowledge on Early Childhood Development. *Int J Pediatr.* 2018;6(3):7371–7382. 10.22038/ijp.2017.27419.2364

[30] Tomopoulos S Dreyer BP Tamis-LeMonda C: Books, toys, parent-child interaction, and development in young Latino children. *Ambul Pediatr.* 2006;6(2):72–8. 10.1016/j.ambp.2005.10.001 16530142

[31] Mol SE Bus AG: To read or not to read: a meta-analysis of print exposure from infancy to early adulthood. *Psychol Bull.* 2011;137(2):267–96. 10.1037/a0021890 21219054

[32] Moore M Wade B: Bookstart: A qualitative evaluation. *Educ Rev.* 2003;55(1):3–13. 10.1080/00131910303250

[33] Squires J Potter L Bricker D: The ASQ user's guide for the Ages & Stages Questionnaires: A parent-completed, child-monitoring system.Paul H Brookes Publishing,1995 Reference Source

[34] Yu LM Hey E Doyle LW: Evaluation of the Ages and Stages Questionnaires in identifying children with neurosensory disability in the Magpie Trial follow‐up study. *Acta Paediatr.* 2007;96(12):1803–8. 10.1111/j.1651-2227.2007.00517.x 17971191

[35] Kapci EG Kucuker S Uslu RI: How applicable are ages and stages questionnaires for use with Turkish children? *Topics Early Child Spec Educ.* 2010;30(3):176–88. 10.1177/0271121410373149

[36] Gollenberg AL Lynch CD Jackson LW: Concurrent validity of the parent-completed Ages and Stages Questionnaires, 2nd Ed. with the Bayley Scales of Infant Development II in a low-risk sample. *Child Care Health Dev.* 2010;36(4):485–90. 10.1111/j.1365-2214.2009.01041.x 20030657

[37] Shahshahani S Vameghi R Azari N: Validity and Reliability Determination of Denver Developmental Screening Test-II in 0–6 Year-Olds in Tehran. *Iran J Pediatr.* 2010;20(3):313–22. 23056723 PMC3446052

[38] Sajedi F Vameghi R Mojembari AK: Standardization and validation of the ASQ developmental disorders screening tool in children of Tehran city. *Tehran Univ Med J.* 2012;70(7):436–446. Reference Source

[39] Chen HY Little R: A test of missing completely at random for generalised estimating equations with missing data. *Biometrika.* 1999;86(1): 1–13. 10.1093/biomet/86.1.1

[40] Shahshahani S Sajedi F Vameghi R: Validity & reliability determination of parents evaluation of developmental status(PEDS) in 4–60 months old children in tehran city. *Iran J Pediatr.* 2014;56–57. Reference Source

[41] Boyle MH Racine Y Georgiades K: The influence of economic development level, household wealth and maternal education on child health in the developing world. *Soc Sci Med.* 2006;63(8):2242–54. 10.1016/j.socscimed.2006.04.034 16790308

[42] Yarosz DJ Barnett WS: Who reads to young children?: Identifying predictors of family reading activities. *Read Psychol.* 2001;22(1):67–81. 10.1080/02702710121153

[43] Kuo AA Franke TM Regalado M: Parent report of reading to young children. *Pediatrics.* 2004;113(6 Suppl):1944–51. 15173465

[44] Karrass J VanDeventer MC Braungart-Rieker JM: Predicting shared parent--child book reading in infancy. *J Fam Psychol.* 2003;17(1):134–46. 10.1037/0893-3200.17.1.134 12666469

[45] Post J Hohmann M: Tender Care and Early Learning: Supporting Infants and Toddlers in Child Care Settings.ERIC; Ypsilanti, MI 48198-2898.2000 Reference Source

[46] Bauer DJ Goldfield BA Reznick JS: Alternative approaches to analyzing individual differences in the rate of early vocabulary development. *Appl Psycholinguist.* 2002;23(3):313–35. 10.1017/S0142716402003016

[47] Braid S Bernstein J: Improved Cognitive Development in Preterm Infants with Shared Book Reading. *Neonatal Netw.* 2015;34(1):10–17. 10.1891/0730-0832.34.1.10 26803041

[48] Farrant BM Zubrick SR: Early vocabulary development: The importance of joint attention and parent-child book reading. *First Lang.* 2012;32(3):343–64. 10.1177/0142723711422626

[49] Brown MI Westerveld MF Trembath D: Promoting language and social communication development in babies through an early storybook reading intervention. *Int J Speech Lang Pathol.* 2017:1–13. 10.1080/17549507.2017.1406988 29243504

[50] Sajedi F Habibi E Hatamizadeh N: Dataset 1 in: Early storybook reading and childhood development: A cross-sectional study in Iran. *F1000Research.* 2018 10.5256/f1000research.14078.d198657 PMC6092897 30135726

