# Supplementary material for: Early storybook reading and childhood development: A cross-sectional study in Iran
Source: F1000Res. 2018 Mar 29;7:411. [Version 1] doi: 10.12688/f1000research.14078.1 (PMC6092897; doi:10.12688/f1000research.14078.1)
Supplement: Supplementary file 2 [file f1000research-7-15314-s0001.tgz › 4c520402-41e3-45f4-ac3c-bff3c41d0151.zip!/Supplementary File 1/English translation.docx]

In the name of God

University of Social Welfare and

Rehabilitation Sciences, Iran

**Early story book reading and early childhood development**

**Questionnaire**

Code: Date:

The name of Health Center: …………………………….. Location: west: 🞎	East:🞎

**A-Demographic** **Questionnaire**

- Sex: Girl 🞎	Boy 🞎
- Childe age (month): …………
- Delivery Type: Caesarian Section 🞎	Vaginal 🞎
- Birth Weight (Gr): …………
- Birth Rank: ………………
- Number of Sibilings: …………….
- Gestational Age (Week): ……………
- Mother Age: ……….
- Father Age: ……….
- Mother Job: Housewife 🞎

Government 🞎

Privet 🞎

Other 🞎

- Father Job: Unemployed 🞎

Government 🞎

Privet 🞎

Other 🞎

- The level of mother education: Under Diploma 🞎

Diploma 🞎

Associate and BS 🞎

MS and above 🞎

- The level of father education: Under Diploma 🞎

Diploma 🞎

Associate and BS 🞎

MS and above 🞎

- Child Caregivers: Mother 🞎	Father 🞎	Grandparents 🞎	Others 🞎
- Monthly Income (Rial): ………………………..
- Income Satisfaction: Yes 🞎	No 🞎

**B- Checklist of Early Storybook Reading**

-How many children book do you have at your home? ……………………….

-How long you or another person read a story book to your child per day (min)? ………………..

-How long you or another person read a story book to your child per day (min)? ………………..

-How long you or another person tell a story for your child per day (min)? ………………..

-How long you or another person sing for your child per day (min)? ………………..
