# Supplementary material for: Early storybook reading and childhood development: A cross-sectional study in Iran
Source: F1000Res. 2018 Mar 29;7:411. [Version 1] doi: 10.12688/f1000research.14078.1 (PMC6092897; doi:10.12688/f1000research.14078.1)
Supplement: Supplementary file 2 [file f1000research-7-15314-s0001.tgz › 4c520402-41e3-45f4-ac3c-bff3c41d0151.zip!/Supplementary File 1/Persian Questionnaire.docx]

به نام خدا

دانشگاه علوم بهزیستی و توانبخشی

«پرسشنامه بررسی وضعیت کتاب خواندن در کودکان و رابطه آن با تکامل دوران ابتدای کودکی»^[1]^

کد: تاریخ:

نام مرکز بهداشتی درمانی: موقعیت: غرب 🞎	شرق🞎

**الف) پرسشنامه جمعیت شناختی**

جنس: دختر 🞎	پسر🞎

سن کودک ( ماه) : .............

نوع زایمان : سزارین 🞎	طبیعی 🞎

وزن تولد ( برحسب گرم ): ...................

رتبه تول : .....................

سن جنینی ( برحسب هفته ): .........................

سن مادر : .......................

سن پدر : .......................

شغل مادر: خانه دار 🞎

شغل دولتی 🞎

شغل خصوصی 🞎

سایر 🞎

شغل پدر: بیکار 🞎

شغل دولتی 🞎

شغل خصوصی 🞎

سایر 🞎

سطح تحصیلات مادر : زیر دیپلم 🞎

دیپلم 🞎

فوق دیپلم و لیسانس 🞎

فوق لیسانس و بالاتر 🞎

سطح تحصیلات پدر : زیر دیپلم 🞎

دیپلم 🞎

فوق دیپلم و لیسانس 🞎

فوق لیسانس و بالاتر 🞎

مراقبین کودک شما شامل چه کسانی می باشد؟

مادر	🞎

پدر 🞎

مادربزرگ و پدربزرگ 🞎

سایر افراد 🞎

میزان درآمد ماهیانه خانوده :..........................

آیا از درآمد خود رضایت دارید؟	بلی 🞎	خیر🞎

**ب) چک لیست وضعیت کتابخوانی برای کودک**

- کودک شما چه تعداد کتاب داستان در خانه دارد؟ ........................

-­شما یا فرد دیگری چند دقیقه در روز برای کودک کتاب داستان می خوانید؟ .......................

-­شما یا فرد دیگری چند دقیقه در روز برای کودک قصه می گویید؟ .......................

-­شما یا فرد دیگری چند دقیقه در روز برای کودک شعر یا آواز می خوانید؟ ....................

**B- Checklist of Early Storybook Reading**

-How many children book do you have at your home? ……………………….

-How long you or another person read a story book to your child per day (min)? ………………..

-How long you or another person read a story book to your child per day (min)? ………………..

-How long you or another person tell a story for your child per day (min)? ………………..

-How long you or another person sing for your child per day (min)? ………………..

1. این پرسشنامه ، قسمتی از پرسشنامه پایان نامه در مورد تکامل دوران ابتدای کودکی می باشد.
