# Supplementary material for: Early storybook reading and childhood development: A cross-sectional study in Iran
Source: F1000Res. 2018 Mar 29;7:411. [Version 1] doi: 10.12688/f1000research.14078.1 (PMC6092897; doi:10.12688/f1000research.14078.1)
Supplement: Peer review history

# Peer review: Referee response for version 1

**DOI:** 10.5256/f1000research.15314.r36960

**Published:** 2018-08-13

**Authors:** Leila Bazrafkan

**Affiliations:** 1 Clinical Education Research Center, Shiraz University of Medical Sciences (SUMS), Shiraz, Iran

The authors of the manuscript conducted a cross-sectional study to assess the state of early childhood development and early storybook reading in Iran. They reported a correlation between Early Storybook Reading (ESR) and early childhood development in communication development domain. I support the conclusion and consider ESR as an environmental stimulation that can promotes early childhood development; it also has a key role in building up the interest in book reading in the future.

  After a detailed review of the report, it could be stated that the study is sufficiently done and reported appropriately. I generally support the main conclusions and find it suitable and acceptable for indexing.

Recommendations for authors:

- It is better to clarify the limitations of the study.
- It seems necessary to specify which version of ASQ was used to assess the state of Early Childhood Development.
- Regarding table1,
  - in data file, columns AD and AE titled as “New mother educate (1=diploma and below, 2=academic)” and “New father educate (1=diploma and below, 2=academic)” were analyzed and reported but in the first column, rows seven and eight, only “Diploma” is mentioned pleas correct it to “Diploma and below”
  - In the first column on row explain the table's footnote that Rials is the Iranian currency

I have read this submission. I believe that I have an appropriate level of expertise to confirm that it is of an acceptable scientific standard.

# Peer review: Referee response for version 1

**DOI:** 10.5256/f1000research.15314.r32641

**Published:** 2018-04-17

**Authors:** Marzieh Rostami Dovom

**Affiliations:** 1 Reproductive Endocrinology Research Center (RERC), Research Institute for Endocrine Sciences, Shahid Beheshti University of Medical Sciences, Tehran, Iran

Thank you for sharing the manuscript entitled “Early storybook reading and childhood development: A cross-sectional study in Iran” with me. I read the paper carefully and find it is credible and appropriate for publication and it doesn’t have any major problem. This is a new topic in Iran and the results of this research can be the base for further research on book reading for children. I think the methods and the results sections are written completely and in clear format. Only as minor comments please follow below points:

  

1. The limitations are more based on suggestions, and the main limitation of the research is not clearly stated.
2. It is suggested that, in the Ethical statement, the cut-off point for the developmental delay should also be mentioned.
3. Some sentences of the article should be edited by a native editor.
4. Add the full form of acronym in the first time appeared in the article. for example: Ages & Stages Questionnaires (ASQ)
5. Did you use a localized standard ASQ questionnaire?
6. In the data set, there are 273 subjects, but data analysis has done for 272 subjects? Is there any reason?
7. Are there any relation between ESD, ECD, and infant sex or type of delivery? My mean is that the gender of infants or even the type of delivery (natural delivery or cesarean section) may have effect on the ESD.  

I have read this submission. I believe that I have an appropriate level of expertise to confirm that it is of an acceptable scientific standard.

# Response

**Published:** 2018-04-18

**Authors:** elham habibi

**Affiliations:** University of Social Welfare and Rehabilitation Sciences, Iran, Iran

Dear Editor/Dear Referee

Many thanks for your email and useful comments from the reviewer, which will certainly help to improve the paper.

***Referring to the comment #6, "In the data set, there are 273 subjects, but data analysis has done for 272 subjects? Is there any reason?" ***, we would like to clarify as below:

With all due respect to the reviewer, we checked the data set (Excel file) again. The first row of the file contains the title of the variables. The number of samples is shown in column B as the code (272 people). Therefore, all samples are considered in the analysis.

We will be pleased to receive and consider any comments from your esteemed referee, which we believe will certainly help to improve the current paper.

Sincerely yours,

E, Habibi
